# Blessing as a health resource: cross-sectional study with elderly residents of rural areas

**DOI:** 10.11606/s1518-8787.2022056003701

**Published:** 2022-08-01

**Authors:** Stephanie Jesien, Luana Patrícia Marmitt, Rodrigo Dalke Meucci

**Affiliations:** I Universidade Federal do Rio Grande Faculdade de Medicina Programa de Pós-Graduação em Saúde Pública Rio Grande RS Brasil Universidade Federal do Rio Grande . Faculdade de Medicina . Programa de Pós-Graduação em Saúde Pública . Rio Grande , RS , Brasil; II Universidade do Oeste de Santa Catarina Programa de Pós-Graduação em Biociências e Saúde Joaçaba SC Brasil Universidade do Oeste de Santa Catarina . Programa de Pós-Graduação em Biociências e Saúde . Joaçaba , SC , Brasil

**Keywords:** Aged, Complementary Therapies, Health Knowledge, Attitudes, Practice, Medicine, Traditional, Rural Health

## Abstract

**OBJECTIVE:**

To estimate the prevalence and factors associated with the search for folk healers for the treatment of health problems among elderly living in the rural area of the city of Rio Grande-RS.

**METHODS:**

Cross-sectional, p opulation-based study with random sampling, carried out in 2017. The outcome was analyzed in three categories (never used/used in the last 12 months/used for more than 12 months). Multinomial logistic regression was used to analyze theassociated factors.

**RESULTS:**

A total of 1,030 elderly individuals were interviewed. The prevalence of demand for folk healers in the last 12 months and for more than 12 months was 9.5% and 15.8%, respectively. In the adjusted analysis, the characteristics associated with the use of a folk healer for more than 12 months were: being in the age group of 80 years or more and having back problems and arthrosis. Following the evangelical religion was identified as a protective factor for using this resource. On the other hand, the demand for blessing in the last year was related to the age group of 70–79 years, following spiritual religions, presence of disease in the last 12 months, back problems and arthrosis, and preference for the use of urgency and emergency services. Being female was associated only with the use for more than 12 months.

**CONCLUSION:**

This study brings an original contribution to a topic poorly evaluated in epidemiological studies, because the knowledge of the frequency and determinants of the search for this type of popular therapy can be used to improve the quality and access to health services offered to the elderly population in rural areas.

## INTRODUCTION

The use of health services happens in the relationship between the need perceived by the individual and the provision of these services. However, in rural areas, access to these services tends to be lower, due to large distances from urban centers, lower supply of health professionals, financial and transportation difficulties, as well as low adherence to healthplans ^1 , 2^ .

Thus, historically, the population living in rural areas sought more affordable alternatives to care for health, among which is the blessing, which is defined as the act of being blessed. It uses rituals with prayers, sometimes also with the use of medicinal plants, and may employ teas, topical pastes or even the act of touching the individual with branches to administer the cure ^3^ . This is an ancient practice, originated from the mixture of cultures and ethnicities (Indians, European and black colonizers) ^4^ , which emerged amid popular Catholicism. Mostly practiced by women and transmitted from generation to generation or received as a “divine gift”, it is characterized by its financial gratuitousness ^5^ , and it is used to treat illnesses, spiritual and emotional problems ^6 , 7^ .

Blessing is part of traditional and complementary medicine, defined as a heterogeneous set of practices, knowledge, and products that do not belong to the scope of conventional medicine ^8^ . In Brazil, several integrative and complementary health practices (PICS) became part of the Unified Health System (SUS) and have been present in ordinances of the Ministry of Health since 2006. Currently, although 29 treatment modalities are recognized by SUS, the services provided by folk healers are not part of the National Policy on Integrative and Complementary Health Practices (PNPICS) ^9^ . However, in the municipality of Sobral-CE, folk healers were incorporated into the teams of health professionals as non-formal health agents that assist in the detection of diseases and referrals to health services ^10^ .

The practice of blessing, which has been applied for a long time, has a little known frequency of use. Most Brazilian studies on the subject are focused on the trajectory and performance of folk healers ^4 , 11^ or bring the search for this type of therapy as a secondary outcome amid the use of integrative practices ^12^ . A cross-sectional study conducted in Minas Gerais with the objective of quantifying alternative complementary medicine reported that 15% of individuals aged 18 years or older had used the services of folk healers at least once in their lives ^7^ . The prevalence was practically the same (14.5%) among adults over 18 years of age in a reference center that carries out treatment for individuals with human immunodeficiency virus (HIV) ^13^ .

Thus, the objective of this study is to estimate the prevalence and factors associated with the use of folk healers’ services for the treatment of health problems in a population of elderly people living in the rural area of the city of Rio Grande, RS.

## METHODS

Cross-sectional population-based study, conducted between April and October 2017, in the rural area of the municipality of Rio Grande-RS. In 2017, the city was estimated to have 209,375 inhabitants, about which 4% of them living in rural areas ^14^ . This study was part of a larger study entitled *“Saúde da população rural rio-grandina”* , which aimed to know the basic health indicators, the morbidity pattern and the use of health services in children under five years old and their mothers, women of childbearing age (15 to 49 years old), and the elderly (60 years old or more). This study focused on the last group, of elderly people aged 60 or older.

The calculation of the sample size necessary to study the prevalence of the use of folk healers’ service considered the following parameters: estimated prevalence of 15% for the use of folk healers ^8^ , confidence level of 95%, margin of error of 2 percentage points and an increase of 10% for losses and refusals, resulting in 638 individuals. For the examination of associated factors, we considered a minimum statistical power of 80%, confidence level of 95%, unexposed/exposed ratio ranging from 54:46 to 80:20, prevalence ratios of 1.7 and the increase of 15% for control of confounding factors. According to these parameters, 874 individuals would be required.

Considering that the rural area of the municipality of Rio Grande consists of 24 census tracts with approximately 8,500 inhabitants distributed in approximately 2,700 permanently inhabited households ^14^ , a systematic random sampling process was used in order to select 80% of the households. This was done by drawing a number between “1” and “5”. The number drawn corresponded to the household considered a “jump”. For example, if the number “3” was drawn, every household number “3” in a sequence of five households was not sampled, that is, it was skipped. These procedures ensured that four out of five households were sampled. The inclusion criteria for participating in the study were: living in the rural area of the city of Rio Grande and being 60 years of age or older. All individuals institutionalized in nursing homes or hospitals were excluded.

The questions used to characterize the outcome were tested from a pre-pilot study, so to test the understanding of the elderly about the questionnaire. After this listening, the following questions were then used: “Have you ever looked for a blessing, a folk healer or a psychic to treat a health problem?”, if so, “When was the last time (years/months) that you looked for a blessing or a folk healer?” We considered positive for the outcome those elderly who claimed to have sought the blessing or healer for health reasons at some time in their lives. Elderly individuals who sought for a folk healer in the last 12 months were asked about: search for a health professional; indication of the search for a health professional by the folk healer; satisfaction with the blessing; payment in cash; and the reason for the search for a folk healer.

In addition, sociodemographic characteristics (gender, age, education, who they live with and religion) and health aspects were investigated: preferred type of health service (basic health unit, doctor’s office, emergency room, others), medical diagnosis in the life of: systemic arterial hypertension, diabetes mellitus, osteoporosis, spinal disease, arthritis or arthrosis. They were also asked if the elderly person had been ill in the last 12 months.

Data collection was performed by trained interviewers, selected and monitored by field supervisors. The electronic questionnaires were applied through tablets using the RedCap ^®^ program ^15^ .

Initially, for data analysis, a description of the studied sample was made. The outcome variable was operationalized into three categories: “never looked”, “searched for it in the last 12 months” and “searched for it more than 12 months ago”. Next, the distribution of the outcome was presented according to the categories of the independent variables. To examine the associated factors, performing crude and adjusted analysis, multinomial regression was used to avoid the loss of information with the simple dichotomization of the outcome.

Thus, the outcome was maintained in three unordered categories. Thus, the category “never looked” (for the blessing) was considered as the reference category. For the adjusted analysis, a hierarchical model was elaborated to control confounding factors ^16^ .

In the first (most distal) level, demographic and socioeconomic variables (gender, age and education) were included; in the second, the variables religion and who they live with. In the third level, the variable related to having become ill in the last 12 months, presence of chronic diseases (systemic arterial hypertension, diabetes mellitus and osteoporosis), spinal disease, arthritis or arthrosis. And finally, at the 4th level (proximal), the type of health service preferred by the elderly. The variables of each level were adjusted at the same level and for the higher level. Those with a p-value < 0.20 were maintained in the model to control for possible confounding factors. The level of significance was 5% for two-tailed tests. The analyses were performed using the statistical package Stata, version 14 ^®^ .

The study was approved by the Research Ethics Committee of the Federal University of Rio Grande, under opinion No. 51/2017, process 23116.009484/2016-26, and the confidentiality of the individual information of the participants was ensured. All the elderly were guaranteed their right to refuse to participate in the research. Those who accepted signed the Free and Informed Consent Form and only then was the questionnaire applied.

## RESULTS

A total of 1,131 elderly were sampled and 1,030 were interviewed, resulting in an 8.9% rate of losses and refusals. In the sample studied, males predominated (55.15%) and 51.4% of respondents were between 60 and 69 years old. Most had up to four years of study and a little more than half (55%) declared themselves Catholics. Of the total population studied, 44.9% had at least one chronic disease (systemic arterial hypertension, diabetes mellitus or osteoporosis), 27.9% reported having some disease in the spine or arthrosis and 19% presented the association of the two health problems. About a quarter (23.8%) reported having been sick in the last year and 8.5% of the elderly reported preference for the use of emergency services when they needed some health service ( Table 1 ).

**Table 1:** Description of the sample of elderly residents of the rural area of Rio Grande, with sociodemographic, social, economic and behavioral variables and the distribution of the prevalence of demand for folk healers between categories. Rio Grande-RS, 2017 (n = 1,030).

	Sample Total	Search for a folk healer			p
		Never looked	Last 12 months	More than 12 months ago	
	% (n)	% (n)	% (n)	% (n)	
		74.7 (756)	9.5 (96)	15.8 (160)	
Sex					0.1
Male	55.1 (568)	54.0 (408)	54.2 (52)	63.1 (101)	
Female	44.9 (462)	46.0 (348)	45.8 (44)	36.9 (59)	
Age (years)					0.003
60–69	51.4 (529)	54.6 (413)	45.8 (44)	42.7 (68)	
70–79	31.8 (326)	29.8 (225)	43.8 (42)	34.0 (54)	
≥ 80	16.8 (173)	15.6 (118)	10.4 (10)	23.3 (37)	
Educational level (years)					0.8
< 1	20.3 (206)	19.8 (148)	16.0 (15)	20.6 (33)	
1–3	35.8 (364)	35.2 (263)	40.4 (38)	37.5 (60)	
4–7	33 (336)	33.2 (248)	34.0 (32)	33.8 (54)	
≥ 8	10.9 (111)	11.8 (88)	9.6 (9)	8.1 (13)	
Living alone					0.6
No	77.4 (797)	76.6 (579)	81.3 (78)	78.7 (126)	
Yes	22.6 (233)	23.4 (177)	18.7 (18)	21.3 (34)	
Religion					< 0.001
Doesn’t have it	21.4 (219)	21.8 (164)	16.7 (16)	20.8 (33)	
Catholic	55.0 (565)	52.6 (396)	61.5 (59)	62.9 (100)	
Evangelical	15.0 (154)	17.9 (135)	6.2 (6)	7.5 (12)	
Spiritualist	8.6 (88)	7.7 (58)	15.6 (15)	8.8 (14)	
Has been sick in the last year					< 0.001
No	76.2 (783)	79 (595)	62.5 (60)	70 (112)	
Yes	23.8 (244)	21 (158)	37.5 (36)	30 (48)	
Chronic diseases ^a^					0.6
0	38.3 (393)	39.8 (300)	34.7 (33)	33.9 (54)	
1	44.9 (460)	43.9 (331)	47.4 (45)	47.8 (76)	
≥ 2	16.7 (171)	16.2 (122)	17.9 (17)	18.2 (29)	
Spine problem and/or arthrosis					< 0.001
None	53.1 (545)	57.6 (435)	34.7 (33)	43.0 (68)	
Spine or arthrosis	27.9 (286)	26.0 (196)	39.0 (37)	30.4 (48)	
Spine and arthrosis	19.0 (195)	16.4 (124)	26.3 (25)	26.6 (42)	
Place they seek health care					0.03
Outpatient Services	91.5 (937)	92.8 (699)	86.3 (82)	88.6 (140)	
Urgency and emergency services	8.5 (87)	7.2 (54)	13.7 (13)	11.4 (18)	

Note: the variable with the highest number of ignored values was education (n = 13).

Spiritualists: category created uniting the religions candomblé, umbanda and spiritualism.

^a^ Chronic diseases: hypertension, diabetes mellitus and osteoporosis.

The prevalence of use of folk healers’ services in the last 12 months and more than 12 months ago was 9.5% and 15.8%, respectively ( Table 1 ). The main characteristics that differed from those that never sought a blessing from those that did (in the last 12 months or more than 12 months prior) were: age; higher proportion of evangelicals; lower proportion of self-reported back problems and/or arthrosis and disease in the last year; and greater use of outpatient services ( Table 1 ).

Elderly people aged 70–79 years (PR = 1.75; 95%CI: 1.11–2.76), spiritualists – spiritists, candomblecists, umbandists (PR = 2.47; 95%CI: 1.13–5.36) –, who became ill in the last year (PR = 1.96; 95%CI: 1.23–3.14), who had some problem in the spine and arthrosis (PR = 2.71; 95%CI: 1.48–4.95), and those who prefer to seek care in urgency and emergency services (PR = 2.26; 95%CI: 1.14–4.45) ( Table 2 ) presented higher occurrence of seeking folk healers’ services in th last 12 months.

**Table 2:** Rate of crude or adjusted prevalence for associations between the use of folk healers in the last year or more than one year prior and independent variables. Sample of elderly residents of the rural area. Rio Grande-RS, 2017 (n = 1,012).

Level	Variable	Folk healer in the last 12 months				Folk healer more than 12 months ago			
		Crude PR (95%CI)	p	Adjusted PR (95%CI)	p	Crude PR (95%CI)	p	Adjusted PR (95%CI)	p
1st	Sex		0.971		0.890		0.035		0.035
	Male	1.00		1.00		1.00		1.00	
	Female	0.99 (0.65–1.52)		1.03 (0.67–1.58)		0.68 (0.48–0.97)		0.68 (0.48–0.97)	
	Age (years)		0.001		0.001		0.003 ^a^		0.002 ^a^
	60–69	1.00		1.00		1.00		1.00	
	70–79	1.75 (1.11–2.76)		1.75 (1.11–2.76)		1.46 (0.98–2.16)		1.43 (0.97–2.12)	
	≥ 80	0.80 (0.39–1.63)		0.79 (0.39–1.63)		1.90 (1.21–2.99)		1.95 (1.24–3.06)	
	Living alone		0.307		0.275		0.555		0.368
	No	1.00		1.00		1.00		1.00	
	Yes	0.75 (0.44–1.29)		0.74 (0.43–1.27)		0.88 (0.58–1.34)		0.82 (0.54–1.25)	
	Educational level (years)		0.680		0.767		0.784		0.810
	< 1	1.00		1.00		1.00		1.00	
	1–3	1.43 (0.76–2.68)		1.40 (0.74–2.64)		1.02 (0.64–1.64)		1.12 (0.69–1.80)	
	4–7	1.27 (0.67–2.43)		1.28 (0.66–2.46)		0.98 (0.61–1.58)		1.07 (0.66–1.74)	
	≥ 8	1.01 (0.42–2.40)		1.06 (0.44–2.56)		0.66 (0.33–1.33)		0.72 (0.35–1.49)	
2nd	Religion		0.001		0.003		< 0.001		0.011
	Doesn’t have it	1.00		1.00		1.00		1.00	
	Catholic	1.53 (0.85–2.73)		1.46 (0.81–2.63)		1.25 (0.81–1.94)		1.34 (0.87–2.09)	
	Evangelical	0.46 (0.17–1.20)		0.45 (0.17–1.19)		0.44 (0.22–0.89)		0.46 (0.22–0.96)	
	Spiritualist	2.65 (1.23–5.70)		2.47 (1.13–5.36)		1.20 (0.60–2.40)		1.31 (0.65–2.65)	
3rd	Has been sick in the last year		< 0.001		0.005		0.014		0.064
	No	1.00		1.00		1.00		1.00	
	Yes	2.26 (1.44–3.54)		1.96 (1.23–3.14)		1.61 (1.10–2.36)		1.46 (0.98–2.18)	
	Chronic diseases		0.568		0.911		0.514		0.820
	0	1.00		1.00		1.00		1.00	
	1	1.24 (0.77–1.99)		1.00 (0.61–1.65)		1.28 (0.87–1.87)		1.15 (0.77–1.72)	
	≥ 2	1.27 (0.68–2.36)		0.81 (0.42–1.58)		1.32 (0.80–2.17)		1.04 (0.60–1.79)	
	Spine problem and/or arthrosis		< 0.001		< 0.001		< 0.001		< 0.001
	None	1.00		1.00		1.00		1.00	
	Spine or arthrosis	2.49 (1.51–4.10)		2.27 (1.36–3.78)		1.57 (1.04–2.35)		1.55 (1.02–2.37)	
	Spine and arthrosis	2.66 (1.52–4.64)		2.71 (1.48–4.95)		2.17 (1.40–3.34)		2.78 (1.73–4.48)	
4th	Place they seek health care		0.029		0.019		0.076		0.106
	Outpatient Services	1.00		1.00		1.00		1.00	
	Urgency and emergency services	2.05 (1.07–3.92)		2.26 (1.14–4.45)		1.66 (0.95–2.93)		1.62 (0.90–2.91)	

95%CI: 95% confidence interval; crude PR: crude prevalence ratio; adjusted PR: adjusted prevalence ratio.

Note: the group “never looked for blessing” was the reference category of the outcome.

Spiritualists: category created uniting the religions candomblé, umbanda and spiritualism.

^a^ p-value of the linear trend test.

Regarding the demand for folk healers’ services more than twelve months prior, being female (PR = 0.68; 95%CI: 0.48–0.97) and evangelical (PR = 0.46; 95%CI: 0.22–0.96) were factors that contributed to the lack of demand for this service. However, individuals aged 80 years or older (PR = 1.95; 95%CI: 1.24–3.06) and those with back problems and arthrosis (PR = 2.78; 95%CI: 1.73–4.48) were more likely to have sought the service more than a year ago ( Table 2 ).

Table 3 describes some characteristics of the service of folk healers in the last 12 months. About 63% of the individuals also sought a health professional, while 42.7% reported the folk healer recommended seeking care in health services. More than 90% were satisfied with the result of the care received. They were also asked why individuals searched for the blessing and 65% of them answered that it was “because they believe” or “have faith” in the healer’s gifts.

**Table 3:** Characterization of the use of the service provided by folk healers among elderly residents of the rural area to treat health problems in the last year. Rio Grande-RS, 2017 (n = 96).

Variables	% (n)
Searched for a blessing and also for a health professional	63.2 (60)
Folk healer indicated the search for a health professional	42.7 (41)
Was satisfied with the result of the blessing	90.6 (87)
Paid some amount in cash for blessing	6.2 (6)
Went to the folk healer because believes/has faith	65 (61)

## DISCUSSION

This study showed that approximately 10% of the elderly used the services of folk healers in the last 12 months and 16% of them more than 12 months prior to research. Characteristics commonly associated with use in both situations were age, religion, and back problems and/or arthrosis. Report of disease in the last year and preferential search for urgency and emergency services were associated only with use in the last 12 months and the gender variable remained associated only with use more than 12 months ago.

Some studies from different countries have evaluated the prevalence of the use of alternative medicine. In Portugal, the prevalence of the use of these methods was compared between urban and rural residents (25% and 75%, respectively). The most used were: bone manipulation (in both areas), followed by sorcerers (healers) and teas in rural areas, and acupuncture and teas in urban areas ^17^ . In Asia, prevalences of 21.7% and 12.2%, respectively, of the use of healers for the treatment of diarrhea and acute respiratory failure in children under 5 years of age were found ^18^ . In South Africa, a population survey found a prevalence of 2.4% of use of healers among people with some type of mental disorder ^19^ .

In a pilot study conducted with rural workers (18 years or older) born in Mexico who lived in North Carolina, the prevalence of seeking healers was 41% ^20^ . The comparability between the prevalences described in the international literature requires caution, since the various existing curative practices are linked to cultural traditions and have different fundamentals and particularities depending on the region in which they are applied ^21^ .

Studies conducted in Minas Gerais, in the municipality of Montes Claros, evaluated the prevalence of the use of PICS in different populations. Among those over 18 years of age, 15% had used the services of a folk healer at some time in their lives ^7^ . In another study, individuals with common mental disorders used blessing services significantly more, when compared to non-carriers ^12^ . In a convenience sample composed of seropositive individuals, a prevalence of 14.5% was found for the use of folk healers and 78.5% for the use of PICS ^12^ .

The predominance of males in the sample of this study differs from national data that indicate a majority of elderly women in Brazil. However, according to data from the *Instituto Brasileiro de Geografia e Estatística* (IBGE) (2010 Census) for the rural area of Rio Grande, the population of men was larger than that of women, being, therefore, a characteristic of the target population evaluated ^13^ .

Age determines the use of folk healers’ services in different ways, according to the evaluated recall, possibly there is a cohort effect in which individuals aged 70–79 years seek more blessing for their health problems, tending to reduce demand from the age of 80. Studies show that in addition to having worse health conditions, elderly people in this age group living in rural areas tend to use health services less, due to greater barriers to access ^1^ .

Among the determining factors for the choice of health practices is religion. Spiritualist religions (spiritualism, candomblé and umbanda) increase the likelihood of an elderly person using the services of blessings in the last 12 months, a finding that can be explained due to the origin of blessing, which comes from the mixture of European, African, indigenous and Catholic beliefs ^10 , 11^ . The blessings that are part of the health care subcultures (specifically within the popular sector), deal with disorders that affect not only the body, the physical sphere, but are related to social, psychological and/or spiritual issues that affect everyday life as a whole ^22^ . Thus, these individuals not only believe more in the services of blessing, but also tend to have acceptance of these practices within their religions.

Among evangelicals, the lower probability of seeking a blessing in life stems from the fact that Pentecostal religions do not recommend the practice ^5^ . In addition, it is possible that evangelicals may have omitted information that they sought blessings due to the recommendations of their religions. Thus, the prevalence of the outcome in this group may be underestimated.

The significant association between the report of having been sick in the last year and the demand for folk healers in the last 12 months may be due to the barriers found to access health services, such as difficulties in referrals, long waiting time for specialized care and proposed treatments, whether they are medicated or not, and may lead the elderly to seek a more accessible health alternative ^23^ .

Spinal problems and arthrosis were strongly associated with the outcome in both recalls because they are chronic and/or recurrent conditions and often lead to limitation or incapacity. Knowing the use of certain complementary therapies, especially the blessing used by the elderly population, is a concern, since the misuse of these actions can cause damage, sometimes irreversible, to the quality of life of patients. Added to this, there is the occurrence of these difficult management conditions in health services that lead to referrals for examinations and treatments often available only in urban areas and with queues ^4 , 22 , 23^ .

The preference for the use of urgent or emergency services was also associated with a higher probability of looking for the folk healer in the last year. A survey conducted in a hospital in Porto Alegre suggests that many elderly people with chronic diseases choose emergency services over others, showing the need for highly complex care at the time of aggravation ^26^ . In addition, we must consider that, since the folk healer is a close therapeutic option (most of them living in the communities where they provide care) and at no cost, it can be sought as the first treatment option ^4^ . If it is not solved, the consequence of this delay in the search for conventional medicine is the worsening of the disease, leaving the elderly to seek urgent and emergency services, proving the relationship between the demand for health services and the aggravation of a condition or disease. Thus, older adults with this profile also tend to use the services of folk healers more, perhaps because they are a close therapeutic option and have a bond with the community ^4^ .

The high level of satisfaction of the elderly with the therapy offered by the blessing or healer can be influenced by some factors such as accessible language, interaction with the community and the health care offered, without financial retribution. This differentiates this therapy from conventional health consultations ^6^ , which can often result in comfort and strength to cope with the illness of the individual ^24^ .

The findings of this study show that most individuals who used folk healers’ services also sought a health professional. Among those who used a folk healer and health service, a portion had recommendation from the healer to seek a conventional health professional (as reported in other studies) ^4 , 10^ . However, as this is a cross-sectional study, this research is susceptible to reverse causality ^28^ , not allowing to affirm whether these individuals sought the blessing before or after the therapy offered by health professionals. In a qualitative study carried out with folk healers in a municipality located in the interior of Rio Grande do Norte, they recognized the importance of the concomitant use of medicine to their practice, showing their willingness to contribute to the official health system through referrals and recognition of the importance of this system ^4^ .

Every individual has the right to make their own decisions regarding their needs in the health-disease process ^27^ . In qualitative studies, health professionals stated that the use of popular care along with professional health treatment could bring beneficial effects to the health of the sick individual and that this behavior would not necessarily cause a decrease in the search for health services, becoming a complementary alternative to biomedical treatment ^25 , 26^ .

However, it should also be considered that blessing can trigger conflicts regarding medical conduct in cases where the patient resorts to spiritual healing, leaving aside conventional medicine ^27^ . In cases of opposition to the treatment that health institutions propose or delay in seeking qualified health professionals, there may be worsening of the clinical framework or even cause the patient to seek a specialized medical service only in times of severity, increasing the risks to health and also the costs of the service ^29^ . Therefore, the attention of the health team to these other forms of support that the patient can seek is important, recognizing the different healing practices within the same territory.

This research brings an original contribution to a topic very poorly evaluated in population-based studies. Although there is no scientific evidence of its effectiveness in the treatment of several health outcomes, blessing is still used by the population due to belief ^24^ . It is important that the health system can recognize the action of folk healers as a form of health consumption, with an impact not only on an individual being or in small groups, but on the entire population. It should be recognized that the individual is not limited to physiological aspects, he is also endowed with beliefs, habits and customs of his own cultural network ^4^ .

Therefore, it is considered relevant to carry out more studies on the subject in different populations and regions of the country, to deepen the knowledge about the frequency and determinants of the use of blessings, as well as other popular therapies. Seeking to make possible the recognition and identification of the implications of cultural and social beliefs in the health-disease process, expanding care beyond the biological dimension of a population. Thinking about the singularities and particularities of health care used by the population can be a useful tool to improve the quality and access to health services offered to the population, especially in the context of primary care.
