# Supplementary material for: Blessing as a health resource: cross-sectional study with elderly residents of rural areas
Source: Rev Saude Publica. 2022 Aug 1;56:72. doi: 10.11606/s1518-8787.2022056003701 (PMC9388065; doi:10.11606/s1518-8787.2022056003701)
Supplement: Translation

# Translation: Benzeção como recurso em saúde: estudo transversal com idosos moradores de área rural

**Authors:** Stephanie Jesien, Luana Patrícia Marmitt, Rodrigo Dalke Meucci

**Affiliations:** I Brasil Universidade Federal do Rio Grande. Faculdade de Medicina. Programa de Pós-Graduação em Saúde Pública. Rio Grande, RS, Brasil; II Brasil Universidade do Oeste de Santa Catarina. Programa de Pós-Graduação em Biociências e Saúde. Joaçaba, SC, Brasil

**Keywords:** Idoso, Terapias Complementares, Conhecimentos, Atitudes e Prática em Saúde, Medicina Tradicional, Saúde da População Rural

## Abstract

**OBJETIVO:**

Estimar a prevalência e os fatores associados à procura de benzedeiras para tratamento de problemas de saúde entre idosos residentes na área rural do município do Rio Grande-RS.

**MÉTODOS:**

Estudo transversal, de base populacional com amostragem aleatória, realizado no ano de 2017. O desfecho foi analisado em três categorias (nunca usou/usou nos últimos 12 meses/usou há mais de 12 meses). Para análise dos fatores associados foi utilizada regressão logística multinomial.

**RESULTADOS:**

Foram entrevistados 1.030 idosos. As prevalências da procura por benzedeira nos últimos 12 meses e há mais de 12 meses foram de 9,5% e 15,8%, respectivamente. Na análise ajustada, as características associadas à utilização de benzedeira há mais de 12 meses foram: estar na faixa etária de 80 anos ou mais e ter problemas de coluna e artrose. Seguir a religião evangélica foi identificado como fator de proteção para a utilização desse recurso. Já a procura por benzedeira no último ano esteve relacionada com a faixa etária dos 70–79 anos, seguir religiões espiritualistas, presença de doença nos últimos 12 meses, problemas na coluna e artrose e preferência por utilização de serviços de urgência e emergência. Sexo feminino permaneceu associado apenas à utilização há mais de 12 meses.

**CONCLUSÃO:**

Este estudo traz uma contribuição original a um tema pouco avaliado em estudos epidemiológicos, pois o conhecimento da frequência e dos determinantes da busca por esse tipo de terapia popular, pode ser utilizado para melhorar a qualidade e o acesso aos serviços de saúde oferecidos à população idosa de áreas rurais.

## INTRODUÇÃO

A utilização de serviços de saúde se dá na relação entre necessidade percebida pelo indivíduo e a oferta desses serviços. Entretanto, em áreas rurais, o acesso a esses atendimentos tende a ser menor, em função das grandes distâncias de centros urbanos, menor oferta de profissionais de saúde, dificuldades financeiras e de transporte, assim como baixa adesão a planos de saúde ^1 , 2^ .

Assim, historicamente, a população residente de áreas rurais procurou alternativas mais acessíveis para cuidar da saúde, dentre elas, está a benzeção, que é definida como o ato de benzer. Utiliza rituais com rezas, algumas vezes também com o uso de plantas medicinais, podendo fazer uso de chás, pastas de uso tópico ou até mesmo o ato de tocar o indivíduo com ramos para administrar a cura ^3^ . Trata-se de uma prática antiga, originada da mistura de culturas e etnias (índios, colonizadores europeus e negros) ^4^ , que emergiu em meio ao catolicismo popular. Majoritariamente praticada por mulheres e transmitida de geração em geração ou recebida como um “dom divino”, tem como característica sua gratuidade financeira ^5^ , e recorre-se a ela para tratar enfermidades, problemas espirituais e emocionais ^6 , 7^ .

A benzeção faz parte da medicina tradicional e complementar, definida como um conjunto heterogêneo de práticas, saberes e produtos que não pertencem ao escopo da medicina convencional ^8^ . No Brasil, várias práticas integrativas e complementares em saúde (PICS) passaram a integrar o Sistema Único de Saúde (SUS) e estão presentes em portarias do Ministério da Saúde desde 2006. Atualmente, apesar de 29 modalidades de tratamento serem reconhecidas no SUS, os serviços prestados por benzedeiras não integram a Política Nacional de Práticas Integrativas e Complementares em Saúde (PNPICS) ^9^ . Entretanto, no município de Sobral-CE, as benzedeiras foram incorporadas às equipes de profissionais de saúde como agentes não formais de saúde que auxiliam na detecção de doenças e nos encaminhamentos aos serviços de saúde ^10^ .

A prática da benzeção, aplicada desde muito tempo, tem sua frequência de utilização pouco conhecida. A maioria dos estudos brasileiros sobre o assunto é focada na trajetória e atuação das benzedeiras ^4 , 11^ ou trazem a procura por esse tipo de terapia como desfecho secundário em meio a utilização de práticas integrativas ^12^ . Um estudo transversal realizado em Minas Gerais, com objetivo de quantificar a medicina complementar alternativa, relatou que 15% dos indivíduos com 18 anos ou mais tinham utilizado os serviços de benzedeiras alguma vez na vida ^7^ . A prevalência foi praticamente a mesma (14,5%) entre adultos maiores de 18 anos em um centro de referência que realiza tratamento para indivíduos portadores do vírus da imunodeficiência humana (HIV) ^13^ .

Desse modo, o objetivo deste estudo é estimar a prevalência e os fatores associados à utilização dos serviços de benzedeiras para tratamento de problemas de saúde em uma população de idosos residentes na área rural do município do Rio Grande, RS.

## MÉTODOS

Estudo transversal de base populacional, realizado entre abril e outubro de 2017, na área rural do município de Rio Grande-RS. Estima-se que, em 2017, a cidade tivesse 209.375 habitantes, cerca de 4% deles vivendo na zona rural ^14^ . Este estudo fez parte de um estudo maior intitulado “Saúde da população rural rio-grandina”, que teve como objetivo conhecer os indicadores básicos de saúde, o padrão de morbidade e a utilização dos serviços de saúde em crianças menores de cinco anos e suas mães, mulheres em idade fértil (15 a 49 anos) e idosos (60 anos ou mais). Para este trabalho focalizou-se o último grupo, de idosos com 60 anos ou mais.

O cálculo do tamanho amostral necessário ao estudo da prevalência da utilização do serviço de benzedeiras considerou os seguintes parâmetros: prevalência estimada de 15% para utilização de benzedeiras ^8^ , nível de confiança de 95%, margem de erro de 2 pontos percentuais e acréscimo de 10% para perdas e recusas, resultando em 638 indivíduos. Para o exame dos fatores associados, considerou-se um poder estatístico mínimo de 80%, nível de confiança de 95%, razão não expostos/expostos variando entre 54:46 a 80:20, razões de prevalência de 1,7 e o acréscimo de 15% para controle de fatores de confusão. De acordo com esses parâmetros, seriam necessários 874 indivíduos.

Considerando que a área rural do município do Rio Grande é constituída por 24 setores censitários com cerca de 8.500 habitantes distribuídos em aproximadamente 2.700 domicílios permanentemente habitados ^14^ , foi utilizado um processo de amostragem aleatório sistemático de modo a selecionar 80% dos domicílios. Isso foi feito a partir do sorteio de um número entre “1” e “5”. O número sorteado correspondeu ao domicílio considerado pulo. Por exemplo, no caso do número “3” ter sido sorteado, todo domicílio de número “3” de uma sequência de cinco domicílios não era amostrado, ou seja, era pulado. Esses procedimentos garantiram que fossem amostrados quatro em cada cinco domicílios. Os critérios de inclusão para participar do estudo foram: morar na zona rural do município de Rio Grande e ter 60 anos ou mais. Foram excluídos todos os indivíduos institucionalizados em asilos ou hospitais.

As perguntas utilizadas para caracterização do desfecho, foram testadas a partir de um estudo pré-piloto com intuito de testar a compreensão dos idosos acerca do questionário. Após essa escuta, foram então utilizadas as seguintes perguntas: “Alguma vez na vida o(a) sr. (a). procurou por benzedeira, benzedeiro, curandeiro ou rezadeira para tratar de um problema de saúde?”, se sim, “Quando foi a última vez (anos/meses) que o (a) sr. (a). procurou por benzedeira ou curandeiro?” Foi considerado positivo para o desfecho aqueles idosos que afirmaram ter procurado a benzedeira ou curandeiro por motivo de saúde alguma vez na vida. Os idosos que procuraram por benzedeira nos últimos 12 meses foram perguntados sobre: procura por profissional de saúde; indicação da procura por profissional de saúde pela benzedeira; satisfação com a benzeção; pagamento em dinheiro; e o motivo da procura pela benzedeira.

Ademais foram investigadas características sociodemográficas (sexo, idade, escolaridade, com quem reside e religião) e aspectos de saúde: tipo de serviço de saúde de preferência (unidade básica de saúde, consultório médico, pronto atendimento, outras), diagnóstico médico na vida de: hipertensão arterial sistêmica, diabetes mellitus , osteoporose, doença na coluna, artrite ou artrose. Também foi perguntado se o idoso esteve doente nos últimos 12 meses.

A coleta de dados foi realizada por entrevistadoras treinadas, selecionadas e acompanhadas por supervisores de campo. Os questionários eletrônicos foram aplicados por meio de tablets utilizando o programa RedCap ^15^ .

Inicialmente, para a análise dos dados, foi feita uma descrição da amostra estudada. A variável desfecho foi operacionalizada em três categorias: “nunca procurou”, “procurou nos últimos 12 meses” e “procurou há mais de 12 meses”. A seguir, apresentou-se a distribuição do desfecho conforme as categorias das variáveis independentes. Para exame dos fatores associados, realização de análise bruta e ajustada, foi utilizada a regressão multinomial para evitar a perda de informações com a simples dicotomização do desfecho.

Dessa forma, o desfecho foi mantido em três categorias não ordenadas. Com isso, foi considerado a categoria “nunca procurou” (pela benzedeira) como a categoria de referência. Para a análise ajustada, elaborou-se um modelo hierárquico para controle de fatores de confusão ^16^ .

No primeiro nível (mais distal), foram incluídas as variáveis demográficas e socioeconômicas (sexo, idade e escolaridade); no segundo, as variáveis religião e com quem vive. No terceiro nível, a variável relacionada a ter ficado doente nos últimos 12 meses, presença de doenças crônicas (hipertensão arterial sistêmica, diabetes mellitus e osteoporose), doença na coluna, artrite ou artrose. E por fim, no 4° nível (proximal), o tipo de serviço de saúde de preferência do idoso. As variáveis de cada nível ajustaram-se no mesmo nível e para o nível superior. Aquelas com um valor de p < 0,20 foram mantidas no modelo para controlar possíveis fatores de confusão. O nível de significância empregado foi de 5% para testes bicaudais. As análises foram feitas utilizando-se o pacote estatístico Stata, versão 14 ^®^ .

O estudo foi aprovado pelo comitê de ética em pesquisa da Universidade Federal do Rio Grande, sob o parecer Nº 51/2017, processo 23116.009484/2016-26, sendo assegurado o sigilo das informações individuais dos participantes. Todos os idosos tiveram garantido seu direito de recusa a participar da pesquisa. Os que aceitaram, assinaram o Termo de Consentimento Livre e Informado e, só então, foi aplicado o questionário.

## RESULTADOS

Foram amostrados 1.131 idosos tendo sido entrevistados 1.030, o que resultou em uma taxa de 8,9% de perdas e recusas. Na amostra estudada, predominaram indivíduos do sexo masculino (55,15%) e 51,4% dos entrevistados possuíam idade entre 60 e 69 anos. A maioria tinha até quatro anos de estudo e um pouco mais da metade (55%) declararam-se católicos. Do total da população estudada, 44,9% possuíam pelo menos uma doença crônica (hipertensão arterial sistêmica, diabetes mellitus ou osteoporose), 27,9% relatou ter alguma doença na coluna ou artrose e 19% apresentava a associação dos dois problemas de saúde. Cerca de um quarto (23,8%) relatou ter estado doente no último ano e 8,5% dos idosos relataram preferência pela utilização de serviços emergenciais quando necessitavam de algum serviço de saúde ( Tabela 1 ).

**Tabela 1:** Descrição da amostra de idosos residentes da área rural de Rio Grande, com variáveis sociodemográficas, sociais, econômicas e comportamentais e a distribuição da prevalência da procura por benzedeira entre categorias. Rio Grande-RS, 2017 (n = 1.030).

	Total da amostra	Procura por benzedeira			p
		Nunca procurou	Últimos 12 meses	Há mais de 12 meses	
	% (n)	% (n)	% (n)	% (n)	
		74,7 (756)	9,5 (96)	15,8 (160)	
Sexo					0,1
Masculino	55,1 (568)	54,0 (408)	54,2 (52)	63,1 (101)	
Feminino	44,9 (462)	46,0 (348)	45,8 (44)	36,9 (59)	
Idade (anos)					0,003
60–69	51,4 (529)	54,6 (413)	45,8 (44)	42,7 (68)	
70–79	31,8 (326)	29,8 (225)	43,8 (42)	34,0 (54)	
≥ 80	16,8 (173)	15,6 (118)	10,4 (10)	23,3 (37)	
Escolaridade (anos)					0,8
< 1	20,3 (206)	19,8 (148)	16,0 (15)	20,6 (33)	
1–3	35,8 (364)	35,2 (263)	40,4 (38)	37,5 (60)	
4–7	33 (336)	33,2 (248)	34,0 (32)	33,8 (54)	
≥ 8	10,9 (111)	11,8 (88)	9,6 (9)	8,1 (13)	
Vive sozinho					0,6
Não	77,4 (797)	76,6 (579)	81,3 (78)	78,7 (126)	
Sim	22,6 (233)	23,4 (177)	18,7 (18)	21,3 (34)	
Religião					< 0,001
Não tem	21,4 (219)	21,8 (164)	16,7 (16)	20,8 (33)	
Católicos	55,0 (565)	52,6 (396)	61,5 (59)	62,9 (100)	
Evangélicos	15,0 (154)	17,9 (135)	6,2 (6)	7,5 (12)	
Espiritualistas	8,6 (88)	7,7 (58)	15,6 (15)	8,8 (14)	
Esteve doente no último ano					< 0,001
Não	76,2 (783)	79 (595)	62,5 (60)	70 (112)	
Sim	23,8 (244)	21 (158)	37,5 (36)	30 (48)	
Doenças crônicas ^a^					0,6
0	38,3 (393)	39,8 (300)	34,7 (33)	33,9 (54)	
1	44,9 (460)	43,9 (331)	47,4 (45)	47,8 (76)	
≥ 2	16,7 (171)	16,2 (122)	17,9 (17)	18,2 (29)	
Problema na coluna e/ou artrose					< 0,001
Nenhum	53,1 (545)	57,6 (435)	34,7 (33)	43,0 (68)	
Coluna ou artrose	27,9 (286)	26,0 (196)	39,0 (37)	30,4 (48)	
Coluna e artrose	19,0 (195)	16,4 (124)	26,3 (25)	26,6 (42)	
Local que procura atendimento de saúde					0,03
Serviços ambulatoriais	91,5 (937)	92,8 (699)	86,3 (82)	88,6 (140)	
Serviços de urgência e emergência	8,5 (87)	7,2 (54)	13,7 (13)	11,4 (18)	

Nota: a variável com maior número de valores ignorados foi a escolaridade (n = 13).

Espiritualistas: categoria criada unindo as religiões candomblé, umbanda e espiritismo.

^a^ Doenças crônicas: hipertensão arterial, diabetes mellitus e osteoporose.

As prevalências da utilização dos serviços de benzedeira nos últimos 12 meses e há mais de 12 meses foram de 9,5% e 15,8%, respectivamente ( Tabela 1 ). As principais características que diferiram aqueles que nunca procuraram uma benzedeira daqueles que o fizeram alguma vez (nos últimos 12 meses ou há mais de 12 meses) foram: idade; maior proporção de evangélicos; menor proporção de autorrelato de problema na coluna e/ou artrose e de doença no último ano; e maior utilização de serviços ambulatoriais ( Tabela 1 ).

Apresentaram maior ocorrência de recorrer aos serviços de benzedeira nos últimos 12 meses os idosos com idade entre 70–79 anos (RP = 1,75; IC95% 1,11–2,76), espiritualistas – espíritas, candomblecistas, umbandistas (RP = 2,47; IC95% 1,13–5,36) –, que adoeceram no último ano (RP = 1,96; IC95% 1,23–3,14), que tinham algum problema na coluna e artrose (RP = 2,71; IC95% 1,48–4,95), e aqueles que preferem buscar atendimento em serviços de urgência e emergência (RP = 2,26; IC95% 1,14–4,45) ( Tabela 2 ).

**Tabela 2:** Razão de prevalência bruta e ajustada para associações entre utilização da benzedeira no último ano e há mais de um ano e as variáveis independentes. Amostra de idosos residentes da área rural. Rio Grande-RS, 2017 (n = 1.012).

Nível	Variável	Benzedeira nos últimos 12 meses				Benzedeira há mais de 12 meses			
		RP _bruta_ (IC95%)	p	RP _ajustada_ (IC95%)	p	RP _bruta_ (IC95%)	p	RP _ajustada_ (IC95%)	p
1º	Sexo		0,971		0,890		0,035		0,035
	Masculino	1,00		1,00		1,00		1,00	
	Feminino	0,99 (0,65–1,52)		1,03 (0,67–1,58)		0,68 (0,48–0,97)		0,68 (0,48–0,97)	
	Idade (anos)		0,001		0,001		0,003 ^a^		0,002 ^a^
	60–69	1,00		1,00		1,00		1,00	
	70–79	1,75 (1,11–2,76)		1,75 (1,11–2,76)		1,46 (0,98–2,16)		1,43 (0,97–2,12)	
	≥ 80	0,80 (0,39–1,63)		0,79 (0,39–1,63)		1,90 (1,21–2,99)		1,95 (1,24–3,06)	
	Vive sozinho		0,307		0,275		0,555		0,368
	Não	1,00		1,00		1,00		1,00	
	Sim	0,75 (0,44–1,29)		0,74 (0,43–1,27)		0,88 (0,58–1,34)		0,82 (0,54–1,25)	
	Escolaridade (anos)		0,680		0,767		0,784		0,810
	< 1	1,00		1,00		1,00		1,00	
	1–3	1,43 (0,76–2,68)		1,40 (0,74–2,64)		1,02 (0,64–1,64)		1,12 (0,69–1,80)	
	4–7	1,27 (0,67–2,43)		1,28 (0,66–2,46)		0,98 (0,61–1,58)		1,07 (0,66–1,74)	
	≥ 8	1,01 (0,42–2,40)		1,06 (0,44–2,56)		0,66 (0,33–1,33)		0,72 (0,35–1,49)	
2º	Religião		0,001		0,003		< 0,001		0,011
	Não tem	1,00		1,00		1,00		1,00	
	Católicos	1,53 (0,85–2,73)		1,46 (0,81–2,63)		1,25 (0,81–1,94)		1,34 (0,87–2,09)	
	Evangélicos	0,46 (0,17–1,20)		0,45 (0,17–1,19)		0,44 (0,22–0,89)		0,46 (0,22–0,96)	
	Espiritualistas	2,65 (1,23–5,70)		2,47 (1,13–5,36)		1,20 (0,60–2,40)		1,31 (0,65–2,65)	
3º	Esteve doente no último ano		< 0,001		0,005		0,014		0,064
	Não	1,00		1,00		1,00		1,00	
	Sim	2,26 (1,44–3,54)		1,96 (1,23–3,14)		1,61 (1,10–2,36)		1,46 (0,98–2,18)	
	Doenças crônicas		0,568		0,911		0,514		0,820
	0	1,00		1,00		1,00		1,00	
	1	1,24 (0,77–1,99)		1,00 (0,61–1,65)		1,28 (0,87–1,87)		1,15 (0,77–1,72)	
	≥ 2	1,27 (0,68–2,36)		0,81 (0,42–1,58)		1,32 (0,80–2,17)		1,04 (0,60–1,79)	
	Problema na coluna e/ou artrose		< 0,001		< 0,001		< 0,001		< 0,001
	Nenhum	1,00		1,00		1,00		1,00	
	Coluna ou artrose	2,49 (1,51–4,10)		2,27 (1,36–3,78)		1,57 (1,04–2,35)		1,55 (1,02–2,37)	
	Coluna e artrose	2,66 (1,52–4,64)		2,71 (1,48–4,95)		2,17 (1,40–3,34)		2,78 (1,73–4,48)	
4º	Local que procura atendimento de saúde		0,029		0,019		0,076		0,106
	Serviços ambulatoriais	1,00		1,00		1,00		1,00	
	Serviços de urgência e emergência	2,05 (1,07–3,92)		2,26 (1,14–4,45)		1,66 (0,95–2,93)		1,62 (0,90–2,91)	

IC95%: intervalo de confiança de 95%; RP _bruta_: razão de prevalência bruta; RP _ajustada_: razão de prevalência ajustada.

Nota: o grupo “nunca procurou por benzedeira” foi a categoria de referência do desfecho.

Espiritualistas: categoria criada unindo as religiões candomblé, umbanda e espiritismo.

^a^ Valor-p do teste de tendência linear.

Em relação à procura há mais de doze meses pelos serviços de benzedeira, ser do sexo feminino (RP = 0,68; IC95% 0,48–0,97) e evangélico (RP = 0,46; IC95% 0,22–0,96) foram fatores que contribuíram para a não procura pelo serviço de benzedeira. No entanto, os indivíduos com 80 anos ou mais (RP= 1,95; IC95% 1,24–3,06) e aqueles com problema de coluna e artrose (RP = 2,78 IC95% 1,73–4,48) apresentaram maior probabilidade de ter procurado o serviço há mais de um ano ( Tabela 2 ).

A Tabela 3 descreve algumas características do atendimento das benzedeiras nos últimos 12 meses. Cerca de 63% dos indivíduos também procuraram um profissional de saúde, enquanto 42,7% relataram que a benzedeira recomendou a procura por atendimento em serviços de saúde. Mais de 90% estavam satisfeitos com o resultado do atendimento recebido. Também foi perguntado o motivo pelo qual os indivíduos buscaram pela benzedeira e 65% deles responderam que foi “porque acreditam” ou “tem fé” nos dons da benzedeira.

**Tabela 3:** Caracterização da utilização do serviço prestado por benzedeiras entre idosos residentes da área rural para tratar problemas de saúde no último ano. Rio Grande-RS, 2017 (n = 96).

Variáveis	% (n)
Procurou por benzedeira e também por profissional de saúde	63,2 (60)
Benzedeira indicou a procura por profissional da saúde	42,7 (41)
Ficou satisfeito com o resultado da benzedeira	90,6 (87)
Pagou algum valor em dinheiro para benzedeira	6,2 (6)
Foi à benzedeira porque acredita/tem fé	65 (61)

## DISCUSSÃO

Este estudo demonstrou que aproximadamente 10% dos idosos utilizaram os serviços de benzedeiras nos últimos 12 meses e 16% deles há mais de 12 meses. As características comumente associadas à utilização em ambos os recordatórios foram idade, religião e problema de coluna e/ou artrose. Relato de doença no último ano e procura preferencial por serviços de urgência e emergência foram associadas apenas à utilização nos últimos 12 meses e a variável sexo permaneceu associada apenas à utilização há mais de 12 meses.

Alguns estudos de diferentes países avaliaram a prevalência da utilização da medicina alternativa. Em Portugal, foram comparadas as prevalências da utilização desses métodos entre moradores da zona urbana e rural (25% e 75%, respectivamente). Os mais utilizados foram: manipulação óssea (em ambas as áreas), seguido por feiticeiros (curandeiros) e chás nas áreas rurais, e acupuntura e chás nas áreas urbanas ^17^ . Na Ásia, foram encontradas prevalências de 21,7% e 12,2%, respectivamente, da utilização de curandeiros para o tratamento de diarreia e insuficiência respiratória aguda em menores de 5 anos ^18^ . Na África do Sul, em um inquérito populacional, foi encontrada uma prevalência de 2,4% de utilização de curandeiros entre os portadores de algum tipo de transtorno mental ^19^ .

Em um estudo piloto, realizado com trabalhadores rurais (18 anos ou mais) nascidos no México que residiam na Carolina do Norte, a prevalência de procura por curandeiros foi de 41% ^20^ . A comparabilidade entre as prevalências descritas na literatura internacional requer cautela, visto que as diversas práticas curativas existentes estão ligadas a tradições culturais e possuem fundamentos e particularidades diferentes dependendo da região em que são aplicadas ^21^ .

Estudos realizados em Minas Gerais, no município de Montes Claros, avaliaram a prevalência da utilização de PICS em diferentes populações. Entre os maiores de 18 anos, 15% haviam utilizado os serviços de benzedeira alguma vez na vida ^7^ . Em outro estudo, indivíduos portadores de transtornos mentais comuns utilizaram significativamente mais os serviços de uma benzedeira, quando comparados aos não portadores ^12^ . Em uma amostra de conveniência, composta por indivíduos soropositivos, encontrou-se uma prevalência de 14,5% para utilização de benzedeiras e 78,5% para o uso de PICS ^12^ .

A predominância do sexo masculino na amostra deste estudo difere dos dados nacionais que apontam uma maioria de mulheres idosas no Brasil. No entanto, de acordo com os dados do Instituto Brasileiro de Geografia e Estatística (IBGE) (Censo de 2010) para área rural de Rio Grande, a população de homens era maior do que a de mulheres, sendo, portanto, uma característica da população-alvo avaliada ^13^ .

A idade determina a utilização dos serviços de benzedeiras de diferentes modos, conforme o recordatório avaliado, possivelmente, há um efeito de coorte no qual indivíduos com 70–79 anos buscam mais a benzeção para seus problemas de saúde, tendendo a reduzir a procura a partir dos 80 anos. Estudos mostram que além de apresentarem piores condições de saúde, idosos nessa faixa etária residentes em áreas rurais tendem a menor utilização de serviços de saúde, devido a barreiras de acesso maiores ^1^ .

Dentre os fatores determinantes para a escolha por práticas de saúde está a religião. As de cunho espiritualista (espiritismo, candomblé e umbanda), aumentam a probabilidade de um idoso utilizar os serviços de benzedeiras nos últimos 12 meses, achado que pode ser explicado devido à origem da benzeção, que vem da mistura de crenças católicas europeias, africanas e indígenas ^10 , 11^ . As benzedeiras que fazem parte das subculturas de cuidado em saúde (especificamente dentro do setor popular) tratam de perturbações que atingem não apenas o corpo, a esfera física, mas estão relacionadas a questões sociais, psicológicas e/ou espirituais que afetam a vida cotidiana como um todo ^22^ . Desse modo, esses indivíduos não apenas acreditam mais nos serviços das benzedeiras, como tendem a ter aceitação dessas práticas dentro de suas religiões.

Entre os evangélicos, a menor probabilidade de busca por uma benzedeira na vida decorre do fato de religiões pentecostais não recomendarem a prática ^5^ . Além disso, é possível que os evangélicos possam ter omitido a informação de que buscaram benzedeiras devido às recomendações de suas religiões. Assim, a prevalência do desfecho nesse grupo pode estar subestimada.

A associação significativa entre o relato de ter estado doente no último ano e a procura por benzedeiras nos últimos 12 meses pode ser decorrente das barreiras encontradas para acessar os serviços de saúde, como dificuldades para encaminhamentos, elevado tempo de espera para atendimentos especializados e tratamentos propostos, sejam eles medicamentosos ou não, podendo levar os idosos a buscar uma alternativa de saúde mais acessível ^23^ .

Problemas na coluna e artrose se mostraram fortemente associados ao desfecho em ambos os recordatórios por serem condições crônicas e/ou recorrentes e que frequentemente levam à limitação ou incapacidade. Conhecer o uso de certas terapias complementares, em especial a benzeção, utilizada pela população idosa, é uma preocupação, pois o uso indevido dessas ações pode provocar danos, por vezes irreversíveis na qualidade de vida dos pacientes. Somado a isso, temos a ocorrência dessas condições de difícil manejo nos serviços de saúde que levam a encaminhamentos para exames e tratamentos muitas vezes disponíveis apenas em áreas urbanas e com filas de espera ^4 , 22 , 23^ .

A preferência pela utilização de serviços de urgência ou emergência também esteve associada a uma maior probabilidade de procurar pela benzedeira no último ano. Uma pesquisa realizada em um hospital de Porto Alegre sugere que muitos idosos portadores de doenças crônicas optem por serviços de emergência em detrimento de outros, manifestando necessidade de cuidados de alta complexidade no momento da agudização ^26^ . Além disso, devemos considerar que a benzedeira, por ser uma opção terapêutica próxima (maioria delas residentes nas comunidades em que prestam atendimento) e sem custo, podem ser procuradas como primeira opção de tratamento ^4^ . Se não resolutiva, a consequência desse atraso na busca pela medicina convencional é o agravamento da doença, restando ao idoso buscar por serviço de urgência e emergência, comprovando a relação entre a procura pelos serviços de saúde e a agudização de uma condição ou doença. Assim, idosos com esse perfil também tendem a utilizar mais os serviços de benzedeiras, talvez por serem uma opção terapêutica próxima e com vínculo com a comunidade ^4^ .

O alto nível de satisfação dos idosos com a terapia oferecida pela benzedeira ou curandeiro pode ser influenciada por alguns fatores como linguagem acessível, interação com a comunidade e o cuidado em saúde ofertado, sem retribuição financeira. Isso diferencia essa terapia das consultas convencionais de saúde ^6^ , podendo, muitas vezes, resultar em conforto e força para o enfrentamento da enfermidade do indivíduo ^24^ .

Os achados deste estudo mostram que a maioria dos indivíduos que utilizaram serviços de benzedeiras, também procuraram por um profissional de saúde. Dentre os que utilizaram benzedeira e serviço de saúde, uma parcela teve recomendação da própria benzedeira para procurar um profissional de saúde convencional (como relatado em outros estudos) ^4 , 10^ . Entretanto, por se tratar de um estudo de delineamento transversal, esta pesquisa está suscetível à causalidade reversa ^28^ , não permitindo afirmar se esses indivíduos procuraram a benzedeira antes ou depois da terapia ofertada por profissionais da saúde. Em um estudo qualitativo realizado com benzedeiras em um município localizado no interior do Rio Grande do Norte, foi reconhecido pelas benzedeiras a importância do uso concomitante da medicina à sua prática, mostrando-se disponíveis a contribuir com o sistema oficial de saúde por meio de encaminhamentos e reconhecimento da importância desse sistema ^4^ .

Todo indivíduo tem o direito a tomar suas próprias decisões em relação as suas necessidades no processo saúde-doença ^27^ . Em estudos qualitativos, profissionais da saúde afirmaram que a utilização de cuidados populares junto ao tratamento profissional de saúde, poderiam trazer efeitos benéficos à saúde do indivíduo doente e que esse comportamento não causaria necessariamente a diminuição pela busca dos serviços de saúde, tornando-se uma alternativa complementar ao tratamento biomédico ^25 , 26^ .

No entanto, deve-se considerar também que a benzeção pode desencadear conflitos quanto às condutas médicas nos casos em que o paciente recorre à cura espiritual, deixando de lado a medicina convencional ^27^ . Nos casos de oposição ao tratamento que as instituições de saúde propõem ou demora para procura de profissionais da saúde capacitados pode haver agravamento do quadro clínico ou mesmo fazer com que o paciente procure um serviço médico especializado apenas em momentos de gravidade, aumentando os riscos sobre a saúde e também os custos do serviço ^29^ . Por isso, é importante a atenção da equipe de saúde a essas outras formas de apoio que o paciente possa buscar, reconhecendo as distintas práticas de cura dentro de um mesmo território.

Esta investigação traz uma contribuição original a um tema muito pouco avaliado em estudos de base populacional. Ainda que não haja evidências científicas da sua eficácia no tratamento de diversos desfechos em saúde, a benzeção é ainda utilizada pela população em virtude da crença ^24^ . É importante que o sistema de saúde possa reconhecer a ação das benzedeiras como forma de consumo em saúde, com impacto não somente sobre um ser individual ou em pequenos grupos, mas sobre toda a população. Deve-se reconhecer que o indivíduo não se resume a aspectos fisiológicos, é também dotado de crenças, hábitos e costumes de uma rede cultural própria ^4^ .

Considera-se, portanto, relevante a realização de mais estudos sobre o tema em diferentes populações e regiões do país, de modo a aprofundar o conhecimento sobre a frequência e os determinantes da utilização de benzeduras, bem como de outras terapias populares. Procurando tornar possível o reconhecimento e a identificação das implicações de crenças culturais e sociais no processo saúde-doença, ampliando o cuidado para além da dimensão biológica de uma população. Ponderar a respeito das singularidades e particularidades dos cuidados em saúde utilizados pela população pode ser uma ferramenta útil para melhorar a qualidade e o acesso aos serviços de saúde oferecidos à população, sobretudo, no âmbito da atenção básica.
